# O05 Implementation of a national training programme: barriers and facilitators to cascading antimicrobial stewardship training to primary care providers in England

**DOI:** 10.1093/jacamr/dlaf118.005

**Published:** 2025-07-14

**Authors:** Jade Meadows, Ming Lee, Raheelah Ahmad, Helena Wehling, Nina Zhu, Jo Taylor-Egbeyemi, Louise E Smith, Dale Weston, Kieran Hand, Donna M Lecky

**Affiliations:** Primary Care and Interventions Unit, UK Health Security Agency, UK; Primary Care and Interventions Unit, UK Health Security Agency, UK; Imperial College London, NIHR Health Protection Research Unit Healthcare Associated Infections & Antimicrobial Resistance, London, UK; Department of Health Services Research & Management, City St George’s, University of London, London, UK; Behavioural Science and Insights Unit, UK Health Security Agency, UK; Imperial College London, NIHR Health Protection Research Unit Healthcare Associated Infections & Antimicrobial Resistance, London, UK; Behavioural Science and Insights Unit, UK Health Security Agency, UK; Behavioural Science and Insights Unit, UK Health Security Agency, UK; Behavioural Science and Insights Unit, UK Health Security Agency, UK; National Health Service England, UK; Primary Care and Interventions Unit, UK Health Security Agency, UK

## Abstract

**Background:**

To enhance the response to the global threat of antimicrobial resistance, an antimicrobial stewardship training intervention for primary care clinicians was planned to be systematically rolled out nationally in England in 2022. This study aimed to understand stakeholders’ anticipated and experienced barriers and enablers towards implementing this intervention nationally in order to improve implementation of the intervention and form recommendations for future initiatives.

**Methods:**

Two workshops were held, with regionally influential stakeholders at the launch (22 participants) and a year (14 participants) into intervention roll out. Qualitative data were collected using three structured frameworks Theoretical Domains Framework (TDF) to identify barriers and facilitators to implementation, the Expert Recommendations for Implementing Change Framework (ERIC) to identify strategies used or planned for supporting change, and Rose, bud, and thorn to identify what went well, what didn’t go well and any opportunities, and were supplemented with facilitated discussions.

**Results:**

Reported anticipated barriers at launch included lack of secure knowledge and confidence in presenting training content. The anticipated enablers were appointing regional champions and linking the training to clinical professional development. A year later, post implementation, participants reported barriers related to the effort needed to both implement the training, and to keep up with the evolving evidence regarding antimicrobial resistance (AMR). Promoting the benefits of the training and offering financial incentives were cited as facilitators. Barriers that were identified across both workshops were: a lack of capacity, time, and the inability for stakeholders to see the impact of training on antimicrobial resistance rates and clinical outcomes. Purposive strategies most employed in supporting uptake and implementation of antimicrobial stewardship (AMS) interventions more generally, concentrated on educating stakeholders, providing support, and the development of stakeholder relationships. Strategies least employed were financial strategies and changing the regional level infrastructure. Participants also provided suggestions for optimizing the training roll out such as different training formats and refresher training.

**Conclusions:**

Overall, the implementation of a national training programme requires support, both centrally and regionally, to be successful. Regional variation in priorities and practical issues arising that may affect the ability to roll out interventions should be acknowledged when implementing interventions nationwide.

